# Impact of the Healthy Lifestyle Community Program (HLCP-3) on Trimethylamine N-Oxide (TMAO) and Risk Profile Parameters Related to Lifestyle Diseases During the Six Months Following an Intervention Study

**DOI:** 10.3390/nu17020298

**Published:** 2025-01-16

**Authors:** Dima-Karam Nasereddin Alzughayyar, Ragna-Marie Weber, Sarah Husain, Nora Schoch, Heike Englert

**Affiliations:** 1 Faculty for Biology, University of Munster, Schlossplatz 4, D-48149 Munster, Germany; 2 Department of Nutrition, University of Applied Sciences, Corrensstraße 25, D-48149 Munster, Germany; ragna.weber@fh-muenster.de (R.-M.W.); shusain@uni-muenster.de (S.H.); nora.schoch@fh-muenster.de (N.S.); englert@fh-muenster.de (H.E.)

**Keywords:** healthy lifestyle community program (HLCP), trimethylamine N-oxide (TMAO), healthy plant diet index (hPDI), plant diet index (PDI)

## Abstract

Rationale: The dietary components choline, betaine, and L-carnitine are converted by intestinal microbiota into the molecule trimethylamine (TMA). In the human liver, hepatic flavin-containing monooxygenase 3 oxidizes TMA to trimethylamine-N-oxide (TMAO). TMAO is considered a candidate marker for the risk of cardiovascular disease. Methodology: The Healthy Lifestyle Community Program cohort 3 (HLCP-3) intervention was conducted with participants recruited from the general population in Germany (intervention: n = 99; control: n = 48). The intervention included intensive educational workshops, seminars, and coaching activities. The assessment was conducted using a complete case analysis (CCA) of the participants. The intervention was carried out for a ten-week intensive phase and an alumni phase. The interventional program emphasizes adopting a healthy plant-based diet and reducing meat consumption, as adherence to such a diet may lead to lowering TMAO levels. Additionally, it provides general recommendations about physical activity, stress management, and community support. The control group did not receive any intervention. TMAO was evaluated using stable isotope dilution liquid chromatography, and tandem mass spectrometry was used to measure fasting plasma levels of TMAO. Objectives: The present study aimed to determine the impact of the Healthy Lifestyle Community Program (HLCP-3) on risk profiles for lifestyle-related diseases and TMAO plasma levels. Results: Significant decreases in most risk profile parameters were detected, and a non-significant decrease in plasma TMAO levels was observed in the intervention group (0.37 (−1.33; 0.59) µmol/L). Furthermore, for the intervention group, after a six-month follow-up period, there was a significant negative correlation between higher healthy plant diet index (hPDI) scores and a decrease in plasma TMAO (ß = −0.200, *p* = 0.027). Additionally, a significant negative correlation was observed between the TMAO level and the scores for adherence to the plant diet index (PDI) (r = −0.195; *p* = 0.023). Conclusions: HLCP-3 is effective at improving adherence to a plant-based diet and improving risk profile parameters. However, long-term interventions involving stricter dietary programs in the sense of a plant-based diet are recommended if significant decreases in TMAO levels are to be obtained.

## 1. Introduction

The gut microbiota is a significant metabolic organ that is composed of over 10^14^ species of microorganisms [1]. Trimethylamine oxide (TMAO) is a gut microbial metabolite [2]. It is derived directly from the consumption of animal-sourced foods such as fish or seafood, leading to higher TMAO concentrations in the blood [3]. The conversions within the gut microbiome are induced by the microbiota, primarily Firmicutes and Proteobacteria [4,5]. The resultant TMA is absorbed in the colon and metabolized to TMAO by hepatic FMOs (flavin-containing monooxygenase 3 (FMO3), which produces TMAO) [6]. This study focuses on HLCP, which is a community-based intervention program of three cohorts; it combines a behavioral approach on the individual level with an infrastructural approach on the community level. The objective is to enhance participants’ health literacy and drive comprehensive community transformations focused on combating lifestyle-related diseases and reducing reliance on medication, including recommendations of food consumption in the sense of a plant-based diet [7,8]. The clinical prognostic significance of TMAO has been evaluated in multiple meta-analyses, which consistently confirm that elevated circulating TMAO levels are associated with a heightened risk of cardiovascular disease and mortality across diverse populations. A systematic review by Schiattarella et al. showed that each 10 µM increase in the TMAO concentration corresponds to a rise of approximately 7.6% in the relative risk of all-cause mortality [9,10]. Recent studies have linked TMAO to the development of type 2 diabetes [11]; it is also associated with obesity [12]. A study of two cohorts of 1206 prospectively enrolled T2DM patients reported that elevated TMAO levels were associated with increased disease severity [13]. The association between lifestyle diseases and increased TMAO levels is the reason this parameter was chosen for analysis. Additionally, the dietary components of meat and fish, particularly those rich in precursors such as choline, carnitine, and betaine, influence the metabolic activity of gut microbes, thereby impacting TMAO production [1]. Interestingly, both microbiota and diet have a direct influence on lifestyle-related diseases [14]. Moreover, dietary components influence the structure and function of the gastrointestinal microbiota, which in turn affect the synthesis of TMAO [15]. Conversely, lifestyle modifications and interventions are prominent treatment options geared towards influencing TMAO concentrations due to their significant impact on cardio-metabolic health [16]. The literature offers conflicting findings; although certain studies indicate that plant-based diets, including vegetarian and vegan regimens, may have a positive impact on TMAO levels, the evidence remains a subject of debate [13,17]. An interventional study on obese adults by Erickson et al. showed that the combination of a hypocaloric diet and regular exercise has shown promising potential in effectively reducing TMAO levels [18].

In studies comparing omnivorous diets with plant-based diets, circulating levels of TMAO are significantly lower in plant-based diets [15]. Understanding these dietary effects is essential for linking alterations in the gut microbiome to systemic health outcomes. To date, limited research has been conducted on the effects of lifestyle interventions that specifically target a generally healthy population. Therefore, the objective of this study was to assess the impact of a six-month lifestyle intervention on circulating TMAO levels among participants in apparently good health.

## 2. Materials and Methods

### 2.1. Study Design

The present study focuses on the third cohort of the HLCP; it is based on interventional, non-randomized studies designed for the prevention and treatment of lifestyle-related diseases. This study was registered as intervention study in the German Clinical Trials Register (DRKS; reference: DRKS00018846; www.drks.de).

**Figure 1:**
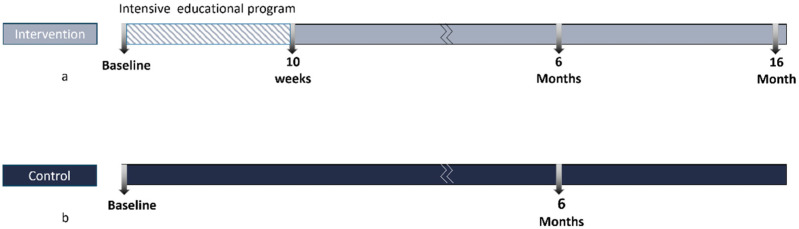
(**a**,**b**) Study design: intervention and control groups (six months).

The lifestyle program served as the intervention group, while the control group received standard care with no intervention at all. Randomization was not possible in this study [8]. To ensure that participants in the control group were not aware of the lifestyle advice given to the intervention group, both groups were recruited from two different small towns. The intervention was conducted in Billerbeck, while the control group was located in Legden. Due to a shortage of staff and time, it was not feasible to recruit new participants for both research arms simultaneously, and the funding was only available for a limited period. As a result, with equal follow-up intervals, the intervention group commenced six months sooner than the control group. Cohort three was not a controlled study, but we utilized the opportunity presented by the availability of the two-point measurements (baseline and six-month follow-up) from the participant samples of the control group of cohort two. This provided the baseline and six-month follow-up measurements. Assessments were made at baseline (March 2019) (t0) and after six months (October 2019) (t2). Baseline (t0) health checks for the control group were completed at the end of (October 2019), and six-month health checks took place in (June 2020) (t2), showed at Figure 1a,b. The study intentionally selected a distant city for the control group to avoid inadvertent exposure to awareness of the program through social connections. Otherwise, the inhabitants of both cities have nearly the same dietary habits and lifestyles. Both groups were statistically comparable, with analyses adjusted for factors such as age, income, gender, educational level, BMI, and smoking. Participants were approximately matched on their BMI and obesity percentage, with no significant differences between groups (*p* > 0.05). This effective matching minimized baseline disparities related to body composition metrics, ensuring a more accurate evaluation of the program’s impact.

### 2.2. Participants

**Figure 2:**
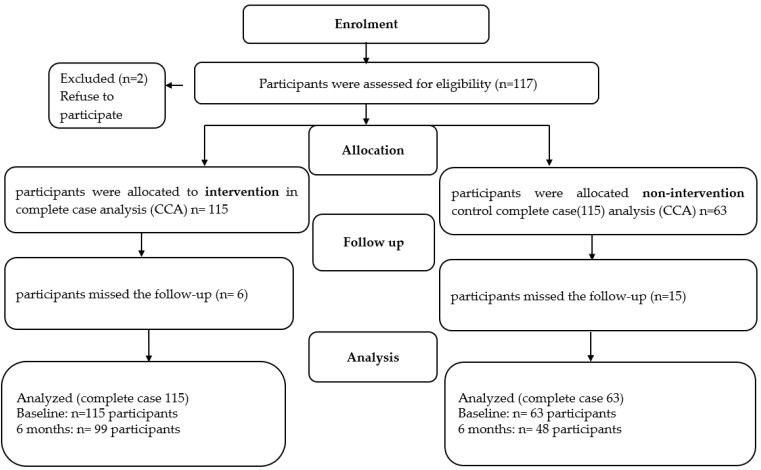
Participant flow chart.

Participants who met the cognitive requirements and were at least 18 years old were recruited, flow chart of participants is shown at Figure 2. The research adhered to the principles outlined in the Declaration of Helsinki, with the necessary approvals from the ethics committees of the Westphalia-Lippe Medical Association and Muenster (Münster, Germany; reference for intervention group: 2019-142-f-S, approved 12 March 2019; reference for control group: 2018-171-f-S, approved 4 April 2018),All participants granted informed assent in writing.

### 2.3. Intervention

The planned intervention in HLCP-3 is a community-based approach to promoting a healthy lifestyle, as previously described [8]. As part of an initial examination (i.e., a health check), an individual risk profile was created for each participant. After ten weeks and six months of follow-up, an analysis was carried out to determine any changes in the parameters over time, as shown in Figure 2.

The intervention was divided into two phases: an intensive phase, which lasted for the initial ten weeks, and an alumni phase, which was less rigorous, lasting from the tenth week until the conclusion of the study. In the intensive phase, 14 seminars and 8 workshops were held. The recommended lifestyle includes at least 30 min of exercise/day and a switch to a healthier diet. The seminar topics centered on a plant-based diet, and the first 10 weeks (the intense phase) of the lifestyle intervention comprised 14 consecutive seminars; the remaining portion of the intervention was made up of monthly seminars, community support motivation, healthy levels of physical activity, and the management of psychological stress, with a strong emphasis on dietary modifications. The seminars encompassed brief practical units, such as cookery demonstrations or sessions with invited visitors, including local general practitioners. In addition to the cookery classes, a guided purchasing tour, archery and table tennis workshops, and a relaxation workshop in nature, participants were granted the opportunity to participate in eight additional workshops in smaller groups (approximately 20 participants each; each workshop lasted one hour).

The dietary recommendations consisted of a shift toward a plant-based, healthy diet, which involved the consumption of a greater variety of plant-based foods (such as fruits, vegetables, legumes, whole grains, nuts, seeds, and healthy oils) and a reduction in the consumption of meat, butter, full-fat dairy, eggs, salt, added sugars, and highly processed foods. Additionally, the participants were advised to reduce alcohol consumption.

### 2.4. Assessment of Parameters

The data collected included laboratory assessments of different targeted blood parameters for the risk profile related to metabolic lifestyle diseases (low-density lipoprotein (LDL), high-density lipoprotein (HDL), cholesterol, triglycerides, glucose, hemoglobin A1c (HbA1c), and TMAO) and the measurements of body weight (kg) and body mass index (BMI). The blood samples were collected by medical professionals in the morning and stored at −80 °C at the hospital of Munster UKM and then transferred in dry ice for analysis at the Laboratory of Mainz. Cardiometabolic risk factors were measured by the Center for Laboratory Medicine of the University Hospital Münster. Serum LDL, HDL, cholesterol, triglycerides, glucose, and HbA1c concentrations were assayed using spectrometry: specifically, UV/VIS photometry on a Roche Cobas c702 analyzer.

Wang et al. [19] employed stable isotope dilution liquid chromatography–tandem mass spectrometry (LC/MS/MS) to measure fasting plasma levels of TMAO in 349 apparently healthy individuals. They reported a median concentration of 3.45 µM (interquartile range 2.25–5.79), with no significant differences between sexes. The TMAO concentration in the plasma was exploratively determined (reference range: 0.73–126 μmol/L) [19]. The method for measuring TMAO in plasma was developed using ANZIMMUN Diagnostics AG in the Mainz Laboratory and certificated by the “Deutsche Akkreditierungsstelle”. The technique is a LC-MSMS method carried out using a Waters Acquity UPLC/TQD, with analysis conducted using a HILIC-column. Apart from the LCMS equipment, no lab automation was used. The cleanup of the samples involved protein precipitation with an organic solvent.

#### Methods for Assessing Adherence to Plant-Based Dietary Patterns

Dietary information was gathered using a semi-quantitative food frequency questionnaire at every health-check appointment (baseline, 10 weeks, 6 months) [7]. Participants were asked about the frequency, on average, of their consumption of a specified portion of 130 food items. These dietary data were used to develop three distinct variants of a plant-based diet for each instance of the food frequency questionnaire health check for the cohort, namely the plant-based diet index (PDI). This group includes all plant-based foods. The healthy plant-based diet index (hPDI) includes healthy plant-based foods, while the uPDI includes less-healthy foods, such as fruit juices, refined grains, potatoes, and sugar-sweetened beverages, in addition to different sources of animal fat. According to the scoring method reported in [20], eighteen food groups were categorized based on nutrient similarities within the broader categories of healthy plant foods, less-healthy plant foods, and animal foods. The groups were divided as follows: cereals and cereal products; vegetables and mushrooms; legumes and legume products; fruit; dairy; meat and fish; potatoes, side dishes, and sauces; cakes, sweets, and snacks; ready-made meals; nuts and seeds; fats and oils; drinks; salt and sugar; and other foods/meals. The adherence to a plant-based diet was assessed using three days of questionnaires, rather than relying on calorie calculations. The PDI, hPDI, and uPDI scores were calculated according to the following equations:

PDI: Total portions of all plant-sourced foods minus total portions of all animal-sourced foods.

hPDI: Total portions of all healthy plant-sourced foods minus the sum of all unhealthy plant-sourced foods and all animal-sourced foods.

uPDI: Total portions of all unhealthy plant-sourced foods minus the sum of all healthy plant-sourced foods and all animal-sourced foods.

The plant food groups were assigned positive scores, while the animal food groups were assigned negative scores. hPDI was developed by assigning positive scores to healthy plant food groups and reverse scores to less healthy plant food groups and animal food groups. In conclusion, the uPDI assigned positive scores to plant food categories that were less healthy.

### 2.5. Statistical Analysis

The statistical analyses were performed using IBM SPSS^®^ Statistics 24 for Windows (Version 25.0, Armonk, NY, USA). The evaluation of the anthropometric and laboratory parameters was carried out as an intention-to-treat analysis (ITT). Analyses were based on unimputed-data-based complete case analysis (CCA). On this basis, the values of the excluded subjects differ in both directions and are compensated on average. Discrete parameters, such as gender, are described by their frequency distribution and evaluated via the chi-square test. The Mann–Whitney U test is used to compare ordinal scaled parameters or responses between the control group and the intervention group. The comparison of the parameters between the measuring times was carried out using the Wilcoxon test (for connected samples). The exact Fisher test was used to assess the dependencies of nominal and binary variables. All the tests mentioned are nonparametric tests that provide reliable results, even in smaller, non-normally distributed samples.

For the analysis of changes from baseline to ten weeks, between-group differences were assessed using a one-way analysis of covariance (ANCOVA). For between-group comparisons of baseline to six-month trajectories, repeated ANCOVA measures were used, using potential confounders as covariates. Bivariate correlations were assessed with Spearman’s rho correlations (two-sided). A sample size calculation was performed based on changes in body weight, as described previously [21]. Fisher’s exact test was used for between-group comparisons of categorical variables. An independent t-test was used for normally distributed continuous variables and a Mann–Whitney U test for non-normally distributed continuous variables. The Shapiro–Wilk test was used to assess data for non-normality (*p* < 0.05 was defined as describing a non-normal distribution).

## 3. Results

### 3.1. Baseline Characteristics

Notably, this study is a component of a comprehensive 16-month study of HLCP-3 [8]. This part of the intervention examined the effect of the intervention on TMAO plasma concentrations. To analyze the changes in TMAO over six months, a total of 178 individuals were initially included, with 115 participants in the intervention group and 63 in the control group). By the end of the six-month period, data from 99 participants in the intervention group and 48 participants in the control group were available for analysis (Figure 2). The intervention group had a higher age, while the baseline TMAO level was not significantly different between the two groups (Table 1). No significant differences were found between groups concerning other baseline characteristics (Table 1). We found no significant difference in terms of risk profile parameters based on baseline values of high total cholesterol (TC), LDL cholesterol (LDL-C), triglycerides (TAG), HbA1c, or low HDL cholesterol (HDL-C). These participants were included in the analyses (body weight; CCA). The participants’ sociodemographic characteristics, their smoker statuses, and the prevalence of overweight and obesity are shown in Table 1. The mean plasma TMAO levels of the baseline for the intervention group and the control group were 9.68 ± 1.25 μmol/L and 8.4 ± 0.62 μmol/L, respectively.

### 3.2. Six-Month Follow-Up from Baseline: Comparison Between the Control Group and the Intervention Group

**Figure 3:**
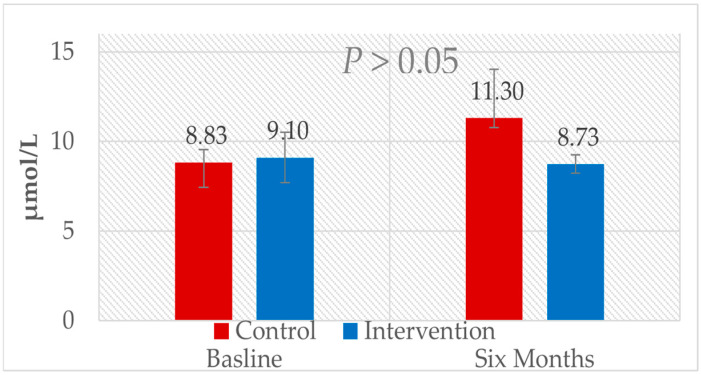
Comparison of TMAO levels between groups after six months of follow up.

**Figure 4:**
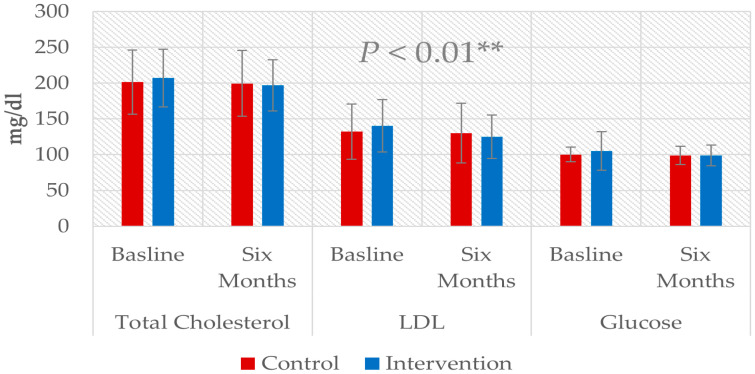
Comparison of risk profile parameters between groups after six months of follow up. ** means *p* ≤ 0.01.

Comparing the six-month follow-up data to the baseline between the two groups showed a non-significant decrease in the level of TMAO for the intervention group, from 9.1 ± 1.4 μmol/L to 8.73 ± 0.52 μmol/L, while the TMAO concentration increased in the control group from 8.83 ± 0.7 μmol/L to 11.3 ± 2.72 μmol/L, as shown in Table 2. The *p* value was >0.05. Significant differences were observed in the risk profile parameters, i.e., cholesterol, LDL-C, LDL-c, and glucose. The *p* value was less than 0.01, as shown in Figure 3 and Figure 4.

#### Comparison of Dietary Scores Between the Intervention and Control Groups After Six Months

The change in dietary scores for the intervention group from the baseline to six months of follow up showed a significant change in terms of adherence to plant-based diets. The PDI increased by 12 points as the mean score changed from 27.5 ± 16.4 to 39.6 ± 18.2. The hPDI points increased by 23 points, with the mean score changing from −7.6 ± 19.9 to 15.7 ± 20.6. The uPDI mean score decreased by seven points, as the mean change was from −38.5 ± 18.6 to −45.7 ± 18.9.

The change in dietary scores for the control group from baseline to six months of follow up showed no adherence to the plant-based diet. The PDI decreased by two points as the mean score changed from 28.3 ± 13.3 to 26 ± 16.3. The hPDI points decreased by six points, with the mean scores changing from −8.6 ± 16.3 to −14.8 ± 20.5. The uPDI mean score increased by two points as the mean changed from −29.2 ± 15 to −31.2 ± 14.9. The *p* value was 0.01 (Table 2).

**Table 2:** Baseline and six-month follow-up measurements in evaluable participants for TMAO and risk factors (IN: n = 109, TMAO n = 90) (Con: n = 48, TMAO n = 48).

Parameters	Group	n	Baseline	Six Months	n	Changes t0 to t2	p WG § Group Effect	p BG # Time Effect	p BG # Interaction Effect (Multivariable-Adjusted)
Body weight, kg	IN	99	80.7 ± 15.1	77.9 ± 15.1	99	−2.8 (−3.49, −2.11)	0.000 ^a^	0.112 ^c^	0.000 ^d^
	Con	48	84.1 ± 17.3	83.9 ± 16.8	47	−0.18 (−1.09, 0.73)	0.698 ^b^		
BMI, kg/m	IN	99	27 ± 4.5	26 ± 4.5	97	−0.98(−0.97, −0.99)	0.000 ^a^	0.057 ^c^	0.000 ^d^
	Con	48	28.2 ± 5.6	28.1 ± 5.7	47	−0.5 (−1.51–0.51)	0.320 ^a^		
TMAO, µmol/L	IN	90	9.1 ± 1.4	8.73 ± 0.52	90	−0.37 (0.59, −1.33)	0.466 ^a^	0.263 ^c^	0.410 ^d^
	Con	48	8.83 ± 0.7	11.3 ± 2.72	46	2.47 (4.18, 0.76)	0.346 ^b^		
PDI, points	IN	99	27.5 ± 16.4	39.6 ± 18.2	98	12.01 (8.05, 15.98)	0.000 ^a^	0.024 ^c^	0.002 ^d^
	Con	48	28.3 ± 13.3	26 ± 16.3	49	−2.29 (−6.01, 1.43)	0.222 ^a^		
hPDI, points	IN	99	−7.6 ± 19.9	15.7 ± 20.6	98	22.77 (18.04, 27.5)	0.000 ^a^	0.000 ^c^	0.000 ^d^
	Con	34	−8.6 ± 16.3	−14.8 ± 20.5	34	−5 (−9.7, −0.31)	0.037 ^a^		
uPDI, points	IN	99	−38.5 ± 18.6	−45.7 ± 18.9	92	−6.59 (−10.79, −2.39)	0.002 ^a^	0.000 ^c^	0.005 ^d^
	Con	48	−29.2 ± 15	−31.2 ± 14.9	49	−1.98 (−6.1, −2.14)	0.339 ^b^		
TC, mg/dL	IN	95	207 ± 40.2	196.7 ± 35.7	95	−9.40 (−14.37, −4.43)	0.000 ^b^	0.532 ^c^	0.029 ^d^
	Con	47	201.3 ± 44.8	199.4 ± 45.9	47	−1.96 (−15.24, 11.32)	0.768 ^b^		
Measured LDL-C, mg/dL	IN	95	140.5 ± 36.6	125.1 ± 30.4	95	−14.54 (−18.89, −10.19)	0.000 ^b^	0.461 ^c^	0.000 ^d^
	Con	47	132.1 ± 38.4	130.1 ± 41.7	47	−2 (−12.83, 8.83)	0.712 ^b^		
Calculated LDL-C, mg/dL	IN	95	123.3 ± 34.4	115.2 ± 29.9	95	−7.42 (−11.53, −3.31)	0.001 ^b^	0.656 ^c^	0.024 ^d^
	Con	47	120.4 ± 36.8	118.1 ± 40.2	47	−2.3 (−13.46, 8.86)	0.680 ^a^		
HDL-C, mg/dL	IN	95	62.2 ± 18.3	59.9 ± 16.3	95	−2.07 (−3.7, 0.45)	0.013 ^b^	0.514 ^c^	0.418 ^d^
	Con	47	57.6 ± 14.9	58.7 ± 16	47	1.06 (−1.77, 3.9)	0.454 ^b^		
TAG, mg/dL	IN	95	107.9 ± 58.8	107.6 ± 53.7	95	0.08 (−7.34, 7.51)	0.982 ^a^	0.905 ^c^	0.474 ^d^
	Con	47	116.5 ± 64.1	111.3 ± 53	47	−5.19 (−18.96, 8.58)	0.452 ^a^		
Glucose, mg/dL	IN	95	104.9 ± 26.9	98.8 ± 14.4	95	−6.01 (−9.6, −2.42)	0.001 ^b^	0.370 ^c^	0.011 ^d^
	Con	47	100 ± 10.2	98.8 ± 13	47	−1.23 (−3.73, 1.26)	0.324 ^a^		
HbA1c, %	IN	95	5.5 ± 0.7	5.3 ± 0.5	95	−1.97 (−2.87, −1.07)	0.000 ^a^	0.056 ^c^	0.056 ^d^
	Con	47	5.6 ± 0.4	5.6 ± 0.4	47	−0.75 (−1.53, 0.03)	0.060 ^a^		
Insulin, µU/mL	IN	95	11 ± 7.9	10.2 ± 6.6	95	−0.68 (−1.8, 0.43)	0.225 ^a^	0.450 ^c^	0.025 ^d^
	Con	47	12.5 ± 9.4	10.8 ± 8.3	47	−1.73 (−3.79, 0.33)	0.097 ^a^		

Values are expressed as means ± SD except for qualitative variables; ANCOVA: one-way analysis of covariance; BMI: body mass index; TMAO: trimethylamine-N-oxide; SD: standard deviation; (t0); baseline; (t2): 6 months; TAG; triglyceride; ; Meas. LDL-C.: Measured LDL-C; Calc. LDL-C: LDL-C Calculated IN: intervention; Con: control group; p WG §: *p*-value within the group effect,: p BG time effect: *p* value between the group; p BG interaction effect: *p* value between the groups. § *p*-value for within-group comparisons by the following tests: ^a^ Wilcoxon test (two-sided); ^b^ paired *t*-test (two-sided); # *p*-value for between-group comparisons by: ^a^ Fisher’s exact test (two-sided); ^b^ Mann–Whitney U test (two-sided); ^c^ independent *t*-test (two-sided); ^d^ repeated measures ANCOVA, adjusted for the baseline, age, and sex mean TMAO, sex, age, smoker status, BMI, total cholesterol, LDL cholesterol, HDL cholesterol, and HbA1c (intervention group: n = 99; control group: n = 47).

### 3.3. Bivariant Correlations

**Figure 5:**
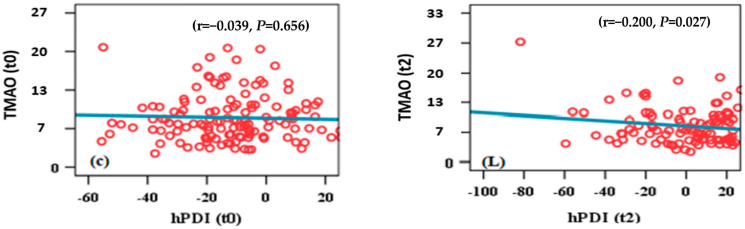
TMAO correlations with healthy plant diet index scores for the intervention group (t0: baseline; t2: 6 months).

**Figure 6:**
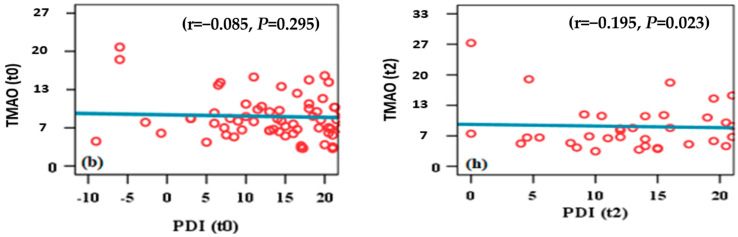
Correlation between TMAO plasma levels and the adherence of participants to the plant-based diet (t0: baseline; t2: 6 months).

At the food-group level, a significant correlation was observed between TMAO levels and adherence to hPDI and PDI within the intervention group; correlations of TMAO and healthy plant diet scores are shown in Figure 5 and Figure 6.

### 3.4. TMAO and hPDI Scatter Plot

Within the intervention group, after six months of follow up (t2), TMAO was negatively correlated (Figure 5) with the baseline values for hPDI (t2); r = −0.200; *p* = 0.027. The red dots highlights the data point representing the subgroup with the highest adherence to a plant-based diet. This group exhibited a notably lower level of TMAO, supporting the hypothesis that intensive adherence to plant-based diets is associated with a significant decrease in TMAO levels. The blue line represents the linear correlation fit, which illustrates the overall trend between the two variables. The reported correlation coefficient (r = −0.039) at baseline, suggests a very weak negative relationship between hPDI and TMAO levels, while the *p*-value (*p* = 0.656) indicates that this relationship is not statistically significant. After six months of follow up the line is showing a negative correlation between the two variables, as reflected by the correlation coefficient (r = − 0.200), with statistical significance (*p* = 0.027).

### 3.5. TMAO and PDI Scatter Plot

In the intervention group and after six months of follow up (t2), TMAO was negatively correlated (Figure 6) with the baseline values (PDI (t2); r = −0.195; *p* = 0.023). The red dots highlights the data point representing the subgroup with the highest adherence to a healthy plant-based diet. This group exhibited a notably lower level of TMAO, supporting the hypothesis that intensive adherence to plant-based diets is associated with a significant decrease in TMAO levels. The blue line, represents the linear correlation fit, which illustrates the overall trend between the two variables. The reported correlation coefficient (r = −0.085) at the baseline, suggests a very weak negative relationship between PDI and TMAO levels, while the *p*-value (*p* = 0.295) indicates that this relationship is not statistically significant. After six months of follow up the line is showing a negative correlation between the two variables, as reflected by the correlation coefficient (r = −0.195). Unlike the previous graph, with statistical significance (*p* = 0.023).

## 4. Discussion

The Healthy Lifestyle Community Program (HLCP) is a healthy lifestyle intervention that initially aims to improve the participants’ risk profile parameters. This part of the study, controlled six months of (cohort-3), focused on observing the impact of the intervention on the different risk profile parameters and TMAO plasma levels.The findings indicated that TMAO levels did not decrease during the six-month follow-up phase of the intervention. The literature presents mixed results; while some studies suggest that plant-based diets, such as vegetarian and vegan diets, may improve TMAO levels, the evidence remains controversial [17,22]. One study involving 34 obese adults preparing for bariatric surgery found that three months of dietary modifications and following exercise recommendations had no significant impact on TMAO levels [23]. In a similar vein, TMAO levels in 220 adults did not change in a second study following nine months of dietary counseling and exercise recommendations [24]. Notably, a hypocaloric diet combined with regular exercise demonstrates potential effectiveness in reducing TMAO levels. However, larger-scale studies are required to validate this finding [18].

These conflicting outcomes may be explained by the complexity of the multiple factors that influence TMAO plasma concentrations, including dietary factors [25]. It is also noteworthy that TMAO levels can be significantly influenced by short-term changes in the consumption of animal protein [26], which means that TMAO is produced over a short period and released into the bloodstream [26]. Another significant factor influencing TMAO levels involves rapid compositional and functional changes in the gut microbiota, which occur over approximately one to five days, in response to either a low-fat, high-fiber plant-based diet or a high-fat, low-fiber animal-based diet [27]. Thus, additional interventional research is necessary to assess the potential relationship between the composition of gut microbiota and the health benefits of adopting a vegan or vegetarian diet. Although there was no significant decrease in TMAO, interestingly, the study’s program succeeded in raising the adherence of the participants to plant-based diets and lowering meat consumption. Plant-based diet scores have primarily been utilized in large cohort studies to support the hypothesis that a higher intake of healthy plant-based foods, concurrent with a lower intake of animal-sourced meals, is related to a decreased risk of CVD [28]. In this study, we observed that the subgroup of participants with stronger adherence to a plant-based diet demonstrated the positive impact of the program’s recommendations, as shown by a decrease in TMAO levels. The correlation between TMAO levels, hPDI, and PDI demonstrates the program’s beneficial impact, thereby providing support for the HLCP-3 hypothesis. On the other hand, other studies findings suggest that the effect of lifestyle interventions will be more efficient in significantly reducing TMAO in the case of long-term adherence to a strict plant-based diet [29,30] or when determining meat consumption [30]. There is convincing evidence that healthy lifestyle choices can measurably improve health [31,32]. One of the main objectives of the HLCP-3 intervention is to increase compliance with healthy food consumption regimes and reduce meat intake in the context of adopting a plant-based diet, without imposing a rigid plant-based diet [8]. As noted in the results, there was notable adherence to plant-based diets. The improvements in the risk profile parameters (total cholesterol, calculated LDL-C, and glucose) indicate the positive impact of the program, supporting our hypothesis that adopting a healthier lifestyle, including a plant-based diet, can lead to a reduction in these risk factors, as various studies have established that the consumption of red meat may contribute to an increased risk of CVD [31]. Promoting healthy lifestyles, both at the individual and population levels, includes encouraging adherence to plant-based dietary recommendations [33]; this may enhance disease prediction and improve body composition [34]. As there is a paucity of prior research investigating the impacts of lifestyle interventions on TMAO, further intervention studies on TMAO are necessary.

## 5. Conclusions

We conclude that the HLCP-3 was effective in improving the risk profile parameters studied. Notably, there was a significant correlation between the decrease in TMAO levels and adherence to a plant-based diet following the program recommendations, even though plasma TMAO levels decreased non-significantly. We recommend longer-term interventions with strict dietary programs. A sustained plant-based diet could be investigated as a potential approach for lowering TMAO levels, given its minimal content of TMAO precursors such as choline and carnitine. Furthermore, to improve adherence in such studies, future efforts might incorporate personalized dietary plans, conduct regular follow-ups, and provide education on the health benefits associated with dietary modifications; these could prove to be effective strategies for changing TMAO levels in relation to cardiovascular diseases.

## 6. Strengths and Limitations

One of the strengths of this study was that it focused on an apparently healthy community of adults with limited major health concerns; most previous studies focused on the role of TMAO as a causative agent of lifestyle diseases or it playing a role as a biomarker [13]. One of the limitations of this study is that it was not possible to conduct a broad analysis of the microbiome and TMAO levels in urine because the high costs of this process exceeded our budget. Additional broad research is required to assess the biological function of trimethylamines, with a specific focus on the function of TMAO in relation to both well-being and illness [4].

## Figures and Tables

**Table 1:** Baseline characteristics of evaluable participants.

Variable	Intervention Group (n = 115)	Control Group (n = 63)	*p*-Value ^#^
Men, n (%)	33 (28.2%)	36 (42.4%)	0.036 ^a^
Age at baseline, years	58.0 ± 8.5	54.7 ± 10.5	0.014 ^b^
Body weight, kg	82.0 ± 15.1	82.9 ± 17.9	0.739 ^c^
BMI, kg/m^2^	27.5 ± 4.5	28.0 ± 5.7	0.526 ^c^
Overweight, n (%)	39 (33.3%)	20 (31.3%)	0.721 ^a^
Obesity, n (%)	32 (27.4%)	25 (39.1%)	0.216 ^a^
TMAO, µmol/L	9.68 ± 1.25	8.4 ± 0.62	0.469 ^b^
Smoker status, n (%)			0.000 ^a^
Never	60 (53.1)	28 (48.3)	
Ex-smoker	46 (10.7)	0 (0.0)	
Smoker	7 (6.2)	30 (51.7)	
Marital status, n (%)			0.590 ^a^
Married	90 (78.9%)	59 (86.8%)	
Partner (unmarried)	7 (6.1%)	3 (4.4%)	
Single (not widowed)	13 (7.1)	13 (7.1)	
Single (widowed)	10 (8.8)	3 (4.4)	

Values are expressed as means ± SD except for qualitative variables. BMI: body mass index; TMAO: trimethylamine-N-oxide. Standard deviation of the mean. ^#^ *p*-value for comparisons between groups by: ^a^ Fisher’s exact test (two-sided). ^b^ Independent *t*-test (two-sided). ^c^ Mann–Whitney U test (two-sided).

## Data Availability

The datasets generated and analyzed during the current study are not publicly available. Data will be available upon reasonable request to the corresponding author.
